# Evaluating the capacity of magnetic ionic liquids for separation and concentration of non-enveloped viral particles and free viral genomic RNA

**DOI:** 10.1007/s00216-024-05662-6

**Published:** 2024-11-28

**Authors:** Sloane Stoufer, Minji Kim, Shashini De Silva, Jared L. Anderson, Byron F. Brehm-Stecher, Matthew D. Moore

**Affiliations:** 1 Department of Food Science, University of Massachusetts, Amherst, MA USA; 2 Department of Chemistry, Iowa State University, Ames, IA USA; 3 Department of Food Science and Human Nutrition, Iowa State University, Ames, IA USA

**Keywords:** Sample preparation, Virus detection, Magnetic ionic liquids, Foodborne viruses, Capture and concentration

## Abstract

Magnetic ionic liquids (MILs) have proven effective as capture reagents for foodborne bacterial pathogens; however, there are currently no published studies regarding their use with foodborne, non-enveloped viruses. In this study, a protocol was evaluated for capture and recovery of bacteriophage MS2, a human norovirus surrogate, and purified viral genomic single stranded RNA (ssRNA) from an aqueous suspension using MILs. Transition metal-based MILs showed similar capture and recovery efficiency for both targets. A rare earth metal-based MIL showed much greater capture efficiency than the transition metal-based MILs, but displayed similar recovery. All tested MILs showed slightly higher capture and recovery efficiency for free RNA in comparison to intact virus, though overall trends were similar, and most MILs could recover both targets at as little as 10^2^ PFU/mL intact MS2 or copies/mL purified RNA. A plaque assay confirmed that contact with MILs did not significantly reduce viral infectivity. Adjusting MIL volume gave no significant changes in capture or recovery, likely due to interplay between volume for the hydrophobic MIL and dispersion. Reducing the elution volume gave a slight increase in recovery, indicating MILs could be used for target enrichment after further optimization. MILs could also capture MS2 from romaine lettuce rinsate at comparable or even higher levels than from pure suspension, though loss in recovery was observed when the rinsate was prepared in an alkaline elution buffer. Overall, these results demonstrate the potential utility of MILs as concentration reagents for foodborne viruses, particularly for in-field applications.

**Graphical Abstract:**

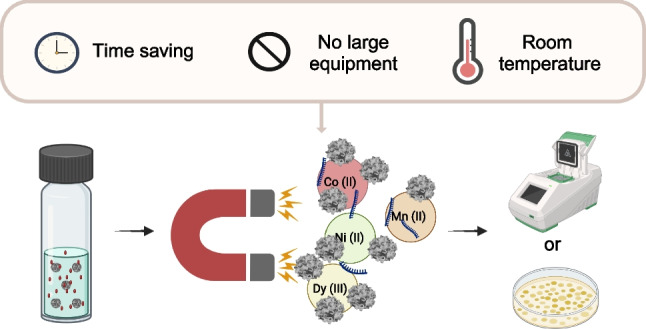

## Introduction

Foodborne illness imposes substantial public health and economic burdens in the USA, as around one in six Americans contract some form of foodborne illness each year. Though there are many potential causative agents of foodborne illness, most cases in the USA are caused by viral pathogens, and of these, nearly all are due to human norovirus (HuNoV). This pathogen is responsible for over 90% of foodborne viral infections by some estimates [1] and results in economic losses of over $2 billion each year [2, 3]. HuNoV is a non-enveloped virus with a positive-sense single-stranded RNA (ssRNA) genome that has a low infectious dose (as little as 1–10 virions) [4, 5] and shows recalcitrance to many surface disinfectants [6–8], making it difficult to control. To prevent and contain HuNoV outbreaks, rapid and early detection of viruses in food and the environment is needed. However, unlike foodborne bacterial pathogens, which are routinely tested for in foods and have accepted levels of a few to zero viable cells [9], there is no requirement of routine testing for viral pathogens in foods. This is partially because current virus detection methods lack sufficient sensitivity and robustness for use with contaminated food samples. Further, most foodborne pathogen testing requires complex equipment that demands specialized training for use and therefore takes place largely at centralized diagnostic laboratories. When accounting for shipping time and potential testing backlogs, it can take days to obtain results, during which time potentially contaminated foods could be widely distributed [10, 11]. Therefore, there is a need for effective separation, concentration, and detection methods for foodborne viruses that can be applied for in-field use in food production facilities without sacrificing sensitivity and specificity.

The “gold standard” method for detection of viral pathogens is RT-qPCR, due to its high sensitivity and specificity [12]. However, this method has some limitations. PCR amplification is vulnerable to inhibition from a variety of components associated with food and clinical samples, such as polysaccharides [13, 14] and heme proteins [14, 15], which can lead to false negative results. This is further complicated by the fact that PCR reactions are performed on microliter-scale assay volumes. Representative food or clinical samples may be orders of magnitude larger, and target pathogens may also be present at low levels and/or unevenly distributed throughout the sample [16, 17]. When testing for bacterial pathogens, these issues can often be mitigated by using cultural enrichment to increase the target concentration to detectable levels while reducing the relative concentration of PCR inhibitors. However, this is rarely feasible for viral pathogens. Isothermal amplification methods are an emerging alternative to PCR that can be adapted for in-field usage, but these methods still require low input volumes, necessitating target concentration or enrichment. To lower the limit of detection for viruses and reduce the risk of false negatives, target separation and concentration is essential.

There are a variety of currently established sample preparation methods for virus detection, but they each have their own limitations. Nonspecific methods, such as polyethylene glycol (PEG) precipitation, ultracentrifugation, and ultrafiltration, exploit physical characteristics of the virus such as their small size and capsid protein characteristics in order to separate them from the sample matrix [18]. PEG precipitation is relatively simple, but requires long incubation times and can co-concentrate matrix-associated proteins. Ultracentrifugation requires costly instrumentation and often co-concentrates potential PCR inhibitors, and ultrafiltration membranes are subject to clogging and fouling, particularly when working with complex food matrices. All of these existing approaches may require additional purification steps and are not well-suited for in-field applications.

Target-specific methods, alternatively, rely on binding ligands such as antibodies, aptamers, or antigens conjugated to magnetic beads to capture the virus. These methods are useful in that they can effectively separate target pathogens from matrix-associated inhibitors and enable their physical enrichment into smaller sample volumes, which aids in downstream detection. However, their high binding specificities can have drawbacks, especially when dealing with foodborne viruses. HuNoV is a very diverse class of viruses with many genogroups and genotypes that are infectious to humans, and certain ligands may display differential affinity for different strains [19, 20]. Additionally, some norovirus genotypes exhibit a relatively high rate of antigenic drift, meaning even ligands that effectively bind many major HuNoV strains may have only a limited window of utility [12].

Aside from the ligands themselves, there are other drawbacks associated with magnetic bead-based sample preparation methods. The production of ligand-functionalized magnetic beads is often costly, making them poor options for high-throughput or large sample volume use. Additionally, these beads require refrigerated storage and have finite shelf lives, which limits their utility in low-resource settings or on-site at farms and food production facilities. In order for routine foodborne virus testing to become feasible, there is a need for rapid target separation and concentration methods that utilize inexpensive shelf-stable reagents and minimize the need for specialized equipment. Such methods should also enable effective capture of intact (infective) viral pathogens, yet remain broadly reactive enough to account for strain variation. This may be possible through the use of magnetic ionic liquids (MILs), whose chemical, physical, and functional properties align with this exacting set of assay requirements.

MILs, a subclass of ionic liquids, are hydrophobic and magnetoactive molten salts with melting points below 100 $^\circ{\rm C}$. While MILs are a broad class of structurally diverse compounds, they generally possess high temperature stabilities and low vapor pressures, may be designed for biocompatibility, and are stable at room temperature over long periods of time [21, 22]. They also have a paramagnetic component, meaning they can be magnetically separated from aqueous suspension. This magnetoactivity, combined with additional cooperative density- and hydrophobicity-based behaviors in aqueous suspension (e.g., post-capture MIL droplet settling and self-aggregation), obviates the need for centrifugation or similar equipment-intensive sample manipulation steps. In short, MILs combine key advantages of magnetic bead-based sample preparation methods, such as speed and minimal equipment requirements, with the physicochemical robustness and cost-effectiveness of non-specific methods.

MILs have previously been used for capture and concentration of *Salmonella* Typhimurium from both simple buffer systems and aqueous food matrices (almond milk, cow’s milk, liquid egg product) [23, 24]. Despite this, it was unknown whether MILs could also bind and concentrate viruses. Given the critical, yet unmet need for effective concentration steps prior to detection of foodborne viruses (all of which are non-enveloped), the purpose of this study was to explore the capacity of MILs for capture and concentration of intact and infectious non-enveloped virus from aqueous suspension. We also investigated the use of MILs for capture and concentration of free genomic viral ssRNA from suspension, both for comparison with intact virus and for evaluation of the potential utility of MILs for extraction of genomic RNA from intact virus, a key preparation step for nucleic acid-based endpoint detection. MILs have been used in previous studies for capture of nucleic acids, including DNA and microRNAs [25–27], and have even been shown to give protection against nucleases in aqueous suspension [28]. However, the ability of MILs to be used in extraction of viral genomic RNA has not yet been reported. Access to a single multitasking reagent capable of capture and concentration of intact foodborne viruses or their ssRNA in suspension and of possibly protecting this RNA against degradation by nucleases would represent a major food safety advance.

## Materials and methods

### MIL synthesis

**Fig. 1:**
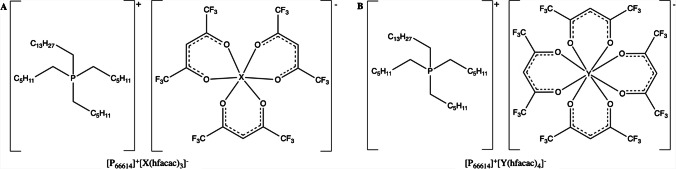
MIL chemical structures.** A** Chemical structures for transition metal-based MILs where X = Co (II), Mn (II), or Ni (II). **B** Chemical structures for rare earth metal-based MILs where Y = Dy (III)

The structures of the MIL formulations used in this study are shown in Fig. 1. Each consisted of a phosphonium cation and a hexafluoroacetyl-acetonate anion with a cobalt (II), manganese (II), nickel (II), or dysprosium (III) metal center and was synthesized according to the method reported by Pierson et al. [29] and detailed within the Supporting Information. After synthesis, MILs were transferred into screw-cap glass vials and kept in a desiccator at room temperature for long-term storage.

### Virus stock preparation

The non-enveloped virus target used was F-specific bacteriophage MS2 (ATCC 15597-B1), which is a commonly used cultivable surrogate for HuNoV given its similar capsid organization and properties [30–32]. MS2 stocks were prepared using a method adapted from Su et al. [33], in which phages were propagated in *Escherichia coli* C-3000 (ATCC 15597) cultured in tryptic soy broth supplemented with 0.1% glucose, 2 mM calcium chloride, and 10 μM thiamine. After propagation, cultures were centrifuged to separate cell debris, aliquoted, and stored at −80 ℃. Enumeration of virus titer was performed using the plaque-count method.

### MIL-based target capture and recovery

**Fig. 2:**
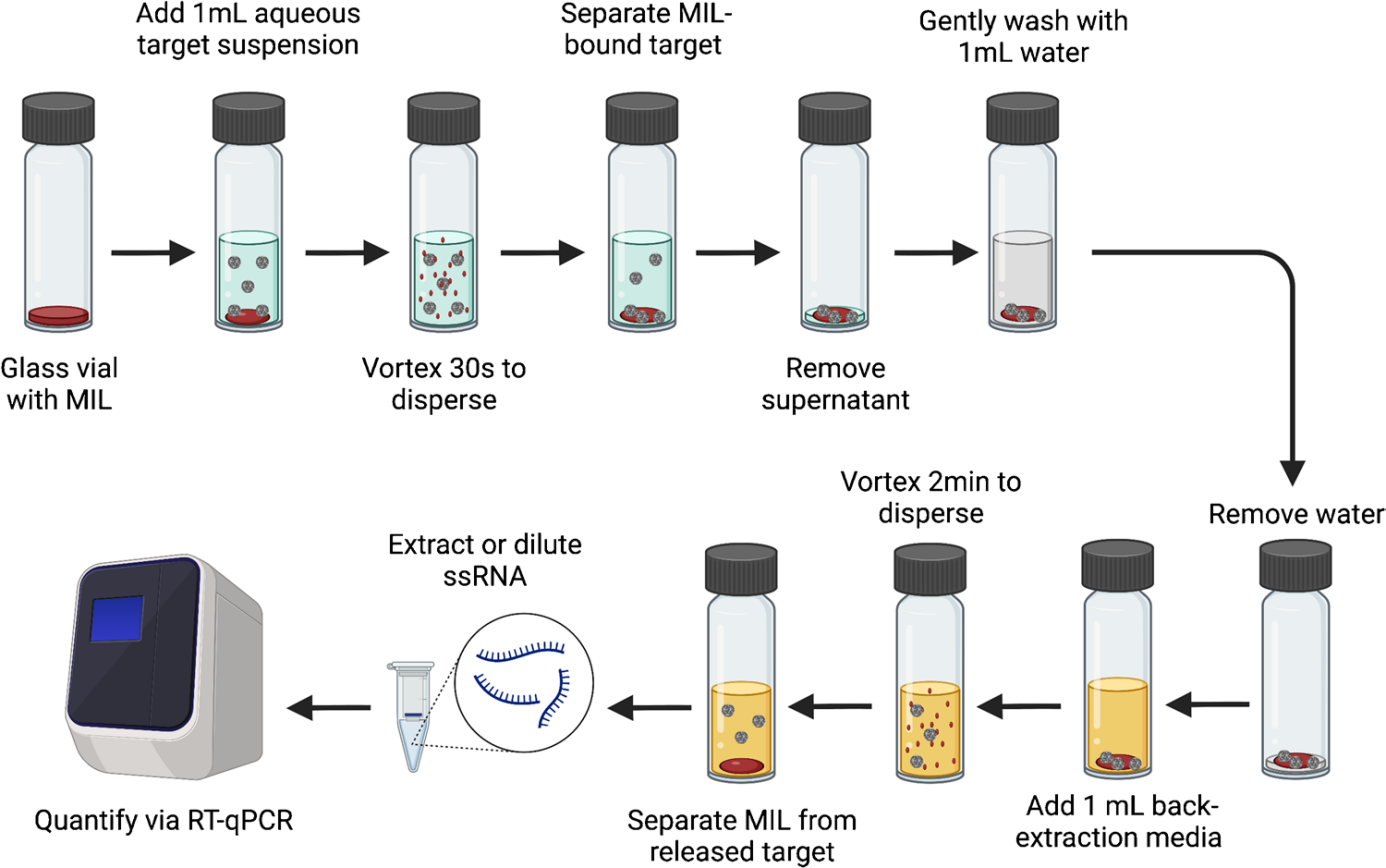
MIL capture and recovery protocol. Basic schematic for MIL-based target capture and recovery, created with BioRender.com

The MIL capture protocol was based on that reported by Clark et al [23] and is outlined in Fig. 2. Briefly, a small volume of MIL (usually 15 μL) was added to a 4-mL screw-cap glass vial that had been degreased by washing with acetone and sterilized by autoclaving. Then, 1 mL of the target suspension, diluted into pH 7.4 phosphate-buffered saline (PBS), was added to the vial, which was then vortexed for 30 s to disperse the MIL droplets using a Fisher Scientific Vortex Mixer on speed 8 (Fisher Scientific, Hampton, NH). The vial then placed on a magnetic rack for 10–15 min to separate the MILs. The supernatant was removed, and 1 mL of nuclease-free water was added, gently mixed, and discarded to remove any additional unbound target. Then, 1 mL of ionically complex elution media (Luria broth with 2X tryptone) was added, and the vial was vortexed again for 2 min to release the bound target. The vial was placed on a magnetic rack for 10–15 min to separate the MILs, and the supernatant was reserved for analysis. When purified ssRNA was used, the sample was diluted tenfold into nuclease-free water before RT-qPCR quantification to reduce inhibition from the elution media. When intact MS2 was used, ssRNA was extracted using the Trizol method before RT-qPCR [34]. Parameters adjusted in this study included target titer, MIL volume, and elution media volume. Unless stated otherwise, the input target titer was 10^5^ PFU/mL for intact MS2 or 10^5^ copies/mL for ssRNA.

### RT-qPCR conditions

RT-qPCR amplification of MS2 ssRNA was performed in a Bio-Rad CFX96 Real Time System (Bio-Rad Systems, Hercules, CA) using the 632F, 708R, and 650P primers and probe targeting the assembly protein gene of MS2 first reported by O’Connell et al. [35]. Reactions were carried out using the NEB Luna Universal One-Step RT-qPCR kit (New England Biolabs, Ipswich, MA) and consisted of a 10-min reverse transcription step at 55 ℃, followed by 10 min at 95 ℃ for enzyme inactivation. Then, 45 cycles of denaturation at 95 ℃ for 15 s and primer annealing/extension at 60 ℃ for 60 s occurred to amplify the target. Each sample was tested in duplicate, and Ct values were averaged and used to extrapolate target copy number by comparing to a standard curve. In brief, tenfold serial dilutions of purified MS2 ssRNA were prepared and assayed in triplicate using the RT-qPCR conditions described. An average Ct value for each dilution was calculated, and the lowest dilution that gave PCR signal in every replicate was defined as one target copy or 0 log10 copies. By plotting average Ct value for each dilution against log10 copies for each dilution, a linear fit (*R*^2^>0.99) was obtained that could be used to calculate ssRNA copy number.

### Calculation of capture and recovery

In each replicate, a no-MIL control vial was included which contained a volume of nuclease-free water equal to the volume of MIL added in the experimental vials and subjected to the same experimental steps (e.g., initial dispersion, water wash and elution). To account for target adherence to the vial itself, initial titer in each replicate was calculated as the log number of copies recovered in the supernatant from the no-MIL control vial after the initial dispersion and separation step. Calculations for capture and recovery were derived from this using the following equations, where *T*_*I*_ represents target copy number found in the supernatant for the no-MIL control vial, *T*_*SUP*_ represents target copy number found in the supernatant for the experimental vials, and *T*_*F*_ represents target copy number recovered from the experimental vials.

1. Percent capture: $$\frac{{T}_{I}{-T}_{SUP}}{{T}_{I}} \times 100$$
2. Percent recovery: $$\frac{{T}_{F}}{{T}_{I}} \times 100$$
3. Log capture: $${Log}_{10}({T}_{I}- {T}_{SUP})$$
4. Log recovery: $${Log}_{10}({T}_{F})$$

### Infectivity testing

To examine the potential impacts of interaction with MILs on MS2 infectivity, a double-layer plaque count method was used to quantify infectious MS2 in post-elution samples [36]. Briefly, bottom agar plates were prepared by pouring 12 mL of 1% tryptic soy agar supplemented with 0.1% glucose and 10 μM thiamine onto 100 × 15 mm petri plates. Top agar consisted of 9 mL 0.5% tryptic soy agar supplemented with 0.1% glucose, 2 mM calcium chloride, and 10 μM thiamine. The top agar was seeded with 0.3 mL of *E. coli* C-3000 grown to OD_600_ of ~0.6 and 0.7 mL of MS2 suspension, poured onto the bottom agar surface, and left to solidify. The plates were incubated overnight (~16 h) at 37 ℃ before plaques were counted.

### Lettuce rinsate testing

To determine if MILs could still function as capture reagents in a more complex suspension, MIL-based capture and concentration was performed on samples of intact MS2 in romaine lettuce rinsate. To prepare rinsate, 25 g of washed romaine lettuce (purchased from a local retailer) and 225 mL of virus elution buffer were combined in a filter stomacher bag and stomached on high speed for 60 s. Intact MS2 was diluted into the prepared lettuce rinsate to 10^5^ PFU/mL and MIL-based capture, and recovery was performed as described above. This method was used as opposed to artificial contamination of whole lettuce leaves in order to minimize variability from factors such as virus degradation during inoculation and efficiency of the viral elution buffers. To test their effects on the MIL-based capture protocol, three different viral elution buffers were used to prepare lettuce rinsate: PBS (pH 7.4), Tris-glycine (100 mM Tris, 50 mM glycine, pH 9.5), and Tris-glycine-beef extract (100 mM Tris, 50 mM glycine, 1% w/v beef extract, pH 9.5).

### Statistical analysis

Two-way analysis of variance (ANOVA) was performed in GraphPad Prism version 9 (GraphPad Software, San Diego, CA) followed by Tukey *post hoc* test for pairwise comparison of means. Differences were considered statistically significant when *p*-value was <0.05.

## Results and discussion

### Capture and recovery of virus and ssRNA targets using various MIL formulations

**Fig. 3:**
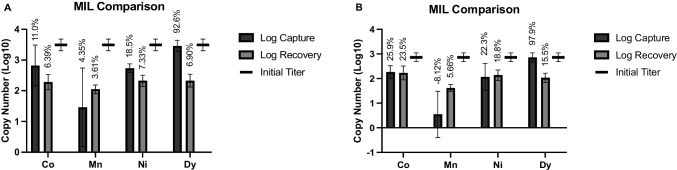
MIL comparison. Separation of **A** intact virus and **B** purified ssRNA from aqueous suspension was performed using different MIL formulations to determine how the different metal centers and anion structures impact capture and recovery for each target. Vertical axis indicates recovered copy number as quantified by RT-qPCR after nucleic acid extraction (intact MS2) or dilution (ssRNA). Percentages for capture and recovery for each condition are indicated above bars

The MIL formulations evaluated in this study had all previously been tested for capture and concentration of bacterial cells from aqueous suspension [23]. However, they had not yet been evaluated with viruses or viral ssRNA. Therefore, as an initial evaluation, each MIL formulation was evaluated for capture and recovery of 10^5^ PFU/mL of intact MS2 or 10^5^ copies/mL of purified ssRNA from aqueous suspension, and results were compared to see how the different formulations performed (Fig. 3). Observed recovery rates were comparable to those achieved with other bead-based methods such as immunomagnetic separation [37] and magnetic silica beads [38, 39], which typically range from 0 to 10% but can be higher depending on the matrix involved. Additionally, no detectable target was recovered in the no-MIL control vials, indicating target recovery was entirely due to the MILs themselves.

No significant differences in recovery were observed between any of the tested MIL formulations for either target, but recoveries ranged slightly higher for purified ssRNA compared with intact MS2 (5.66 $\pm$ 0.82 − 23.5 $\pm$ 5.96% versus 3.61 $\pm$ 0.43 − 7.33 $\pm$ 3.13%, respectively). This may have been due in part to protective effects against RNA degradation. MILs have been previously reported to have protective effects against RNases [40, 41], so it may be that RNA degraded more quickly in the no-MIL control vial than in the experimental vials, causing capture and recovery to appear greater.

There were some notable differences in capture efficiency. The Dy (III)-based MIL, which has a unique anion structure, appeared to show much greater capture affinity for both targets than the transition metal-based MILs, removing 92.6 $\pm$ 0.35% of intact MS2 and 97.9 $\pm$ 1.20% of purified ssRNA from the suspension. However, it did not give correspondingly strong recovery. Conversely, the Mn (II)-based MIL gave no apparent capture in some replicates, meaning target titer in the supernatant after the initial binding step was higher than the input titer. Since this phenomenon persisted across different preparations of the Mn (II)-based MIL, it seems unlikely this was due to nucleic acid contamination. However, it may be that these MILs had some level of antiviral activity. To investigate this, a plaque assay was performed on post-elution MS2 samples.

Previous studies that used these same MIL formulations for capture and concentration of bacterial cells found that, after plate counting, the Dy (III)- and Mn (II)-based MILs gave no viable cells [23, 42]. However, when PCR was performed on samples recovered by the Dy (III)-based MIL, they still observed comparable signal to samples recovered using the Co (II)- and Ni (II)-based MILs [23]. One well-known drawback of nucleic acid-based diagnostics is that once a bacterial cell or viral particle is no longer viable, its genetic material can linger in a suspension and on surfaces for long periods, making it difficult to discriminate between infectious and non-infectious cells/particles in a sample. Since all four MILs tested showed favorable recovery with purified ssRNA, there was a possibility that viral capsid lysis occurred during the MIL binding, and free RNA was recovered from intact MS2 suspensions. Therefore, to evaluate potential effects on viral infectivity and capsid integrity, titers of MS2 samples recovered by each of the tested MIL formulations were quantified by a plaque count assay, as described above (Table 1). Intact MS2 was recovered by each MIL formulation at titers which correlated with the recoveries determined by RT-qPCR. However, greater variation in plaque counts was observed with the Mn (II)- and Dy (III)-based MILs. Since the samples were frozen at −80 ℃ prior to plaque assay enumeration, it is possible that exposure to these MIL formulations made MS2 more vulnerable to damage from freeze-thaw and cold storage. However, none of the MILs appeared to markedly reduce viral infectivity.

**Table 1:** Infectivity testing. MS2 plaque assay was performed on post-elution suspensions to determine if the MILs could successfully recover intact virus without damaging the capsid

MIL	Recovered MS2 titer (PFU/ML)	Standard deviation
Co (II)	1.78 × 10^3^	6.60 × 10^1^
Mn (II)	3.98 × 10^2^	3.12 × 10^2^
Ni (II)	1.38 × 10^3^	3.25 × 10^2^
Dy (III)	2.10 × 10^3^	1.67 × 10^3^

Since the discrepancy between recovery and capture for the Dy (III)-based MIL was not apparently due to target degradation, it is possible that the current elution protocol is insufficient to effectively disrupt the binding between the Dy (III)-based MIL and the target analyte. Further research on alternative elution methods should be performed before attempting to characterize the Dy (III)-based MIL. For this reason, all subsequent experiments were performed using only the transition metal-based MILs.

### Effect of MIL volume and binding efficiency

**Fig. 4:**
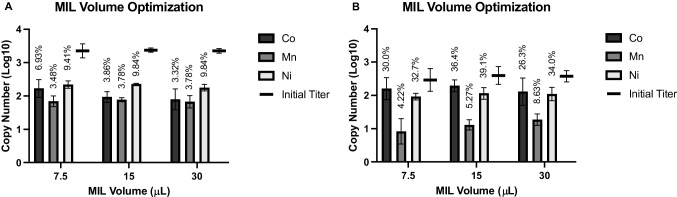
MIL volume optimization. Separation of **A** intact MS2 and **B** purified ssRNA from aqueous suspension was performed using different volumes of MIL (7.5, 15, or 30 μL) per 1 mL of target suspension to determine if MIL volume was limiting target recovery efficiency. Vertical axis indicates recovered copy number as quantified by RT-qPCR after nucleic acid extraction (intact MS2) or dilution (ssRNA). Percent recovery for each condition is indicated above the bars

In order to further optimize the capture protocol and ensure that MIL volume was not a limiting factor in target binding, different volumes of MIL relative to the initial suspension volume were evaluated to determine if this had any impact on target recovery (Fig. 4). No significant differences were observed when 7.5, 15, or 30 μL of MIL was used per 1 mL of target suspension for any of the transition metal-based MILs when used with either intact MS2 or purified ssRNA. It could be that the major limiting factor was MIL droplet formation, which was highly variable due to the nature of the vortex dispersion method and the hydrophobic nature of the MILs themselves [42]. Methods to enhance MIL dispersion would include either adjusting the physical dispersion method or adding solutes to the target suspension to enhance dispersion, which led to formation of smaller MIL droplets after vortex dispersion in previous studies by Hice et al. [42]. This was supported by observations in the present study; by visual, macroscale observation, MIL dispersion appeared to be much greater during target elution with the modified Luria broth than it was in the initial binding step, when only PBS was used. However, future optimization studies would need to balance enhancement of instrumentally observed MIL dispersion with potential impacts on target binding by other buffer components. This would require extensive further study, so for the purposes of this work, 15 μL was used as the MIL volume for all future experiments both to allow for potential improvements in recovery and to maintain consistency and comparability with previous studies [24, 42].

### Effect of target input titer and limit of recovery

**Fig. 5:**
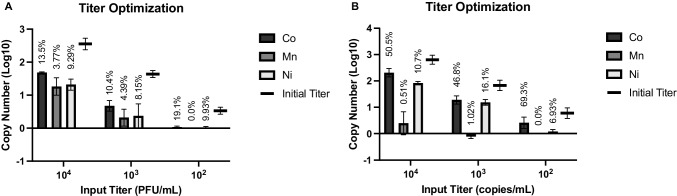
Titer optimization. Suspensions of **A** intact MS2 and **B** purified ssRNA with decreasing titer were tested to evaluate the capture potential and determine limit of detection for the MIL capture and recovery protocol. The vertical axis indicates recovered copy number as quantified by RT-qPCR after nucleic acid extraction (intact MS2) or dilution (ssRNA). Percent recovery for each condition is indicated above the bars

Target input titers ranging from 10^4^ to 10^2^ PFU/mL or copies/mL were then tested to determine if reducing the input titer increased recovery and to identify the limit of recovery for the current protocol. Each of the transition metal-based MILs recovered intact MS2 consistently down to 10^3^ PFU/mL, but at 10^2^ PFU/mL the Co (II)- and Ni (II)-based MILs could not always successfully recover the target, and the Mn (II)-based MIL was unable to recover any MS2 with a 10^2^ PFU/mL input titer. However, the calculated percent recovery showed no significant (*p* > 0.05) changes between each input titer for each of the MILs, ranging from 10.4 $\pm$ 4.44 − 19.1 $\pm$ 18.0% for Co (II), 0.0 − 4.39 $\pm$ 1.56% for Mn (II), and 8.15 $\pm$ 4.17 − 9.93 $\pm$ 17.2% for Ni (II) (Fig. 5). The higher standard deviations for Co (II) and Ni (II) were observed at 10^2^ PFU/mL, when target was only sometimes successfully recovered. Similar results were obtained with purified ssRNA; Co (II) and Ni (II) recovered a similar copy number at all of the tested input titers, but the Mn (II) displayed inconsistent results at 10^3^ copies/mL, and no recovery in any of the replicates at 10^2^ copies/mL was observed. As with intact MS2, percent recovery for ssRNA remained fairly consistent across the different input titers for all MIL formulations. The consistent percent recovery was unexpected, but it is possible that the reduced target titer inhibited MIL dispersion. As mentioned above, MIL dispersion increases with increasing solute concentration, so in a minimal target suspension, residual media in the target itself may act as a solute and help to enhance MIL dispersion at higher titers. This would likely not be an issue when testing an actual food or clinical sample, as these would have many other components that could facilitate MIL dispersion, but it does underscore the need for future studies on methods to enhance MIL dispersion in aqueous target suspension, including the addition of other system components such as solutes or surfactants.

### Effect of elution volume and potential for target enrichment

**Fig. 6:**
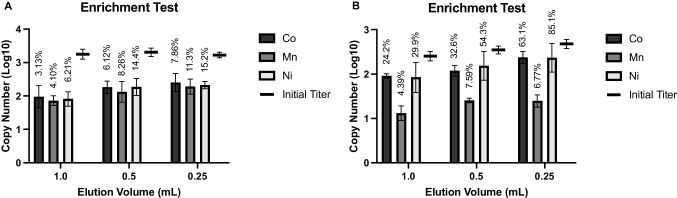
Enrichment test. MIL-bound suspensions of **A** intact MS2 and **B** purified ssRNA were eluted into decreasing volumes of media (1.0, 0.5, and 0.25 mL) to estimate the enrichment capacity of the MIL-based capture and recovery protocol. Vertical axis indicates recovered copy number as quantified by RT-qPCR after nucleic acid extraction (intact MS2) or dilution (ssRNA). Percent recovery for each condition is indicated above the bars

Reduced volumes of media were used in the elution step to provide an estimate of the enrichment capacity of the current protocol. No loss in MS2 recovery was observed when the elution volume was reduced from 1.0 to 0.25 mL (Fig. 6). In fact, a slight (though not statistically significant) increase in both copy number and recovery efficiency was observed as the elution volume decreased with intact MS2 as the target analyte. Even greater enrichment was observed with purified ssRNA; both the Co (II)- and Ni (II)-based MILs displayed significant increases in recovery when 0.25 mL was used as the elution volume compared to 1.0 mL. The elution volume could not be easily reduced below 0.25 mL with the current protocol due to the dimensions of the vials used, but greater enrichment could likely be achieved with lower input titer and higher volumes of initial suspension. Previous studies achieved an enrichment factor over tenfold using the same MIL formulations and a 2-mL initial suspension of 10^4^ PFU/mL bacterial cells, lending support to this idea [23]. Another study found that reducing elution volume from 1 to 0.2 mL helped reduce the limit of detection from 10^4^ CFU/mL of *Salmonella* Typhimurium to 10^3^ CFU/mL [24]. This has significant implications for the practical use of MILs in microbial testing. Larger initial suspension volumes should be evaluated in future work, as one of the primary goals of sample preparation is target enrichment. This is especially relevant for viral pathogens, and particularly for foodborne viruses, as pathogens are typically present at low levels in food samples.

### Capture and recovery from lettuce rinsate

**Fig. 7:**
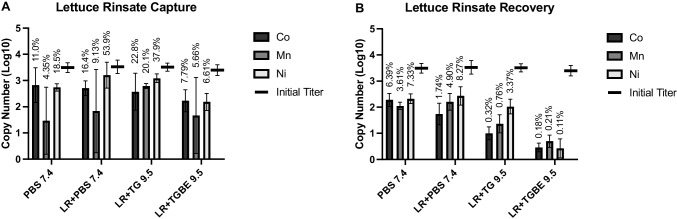
Lettuce rinsate test. Transition metal-based MILs were evaluated for **A** capture and **B** recovery in lettuce rinsate samples prepared in three different viral elution buffers: phosphate-buffered saline pH 7.4 (PBS 7.4), Tris-glycine pH 9.5 (TG 9.5), and Tris-glycine-beef extract pH 9.5 (TGBE 9.5). These were compared to results achieved in pure PBS (PBS 7.4)

Lastly, the MILs were evaluated for their ability to capture and recover MS2 from a sample of lettuce rinsate prepared in different viral elution buffers. For the lettuce rinsate prepared in PBS, a slight increase in capture and recovery was observed for the Mn (II)- and Ni (II)-based MILs compared with the pure MS2 suspension (Fig. 7). Greater MIL dispersion was observed in all the lettuce rinsate samples compared with the pure MS2 suspension, which likely contributed to this increased capture. Similar effects were observed with the Tris-glycine-based elution buffers, with few significant differences in capture efficiency observed compared with the pure suspension. However, all MILs gave comparatively poor recovery efficiency (up to 3.37%) from lettuce rinsate prepared in the Tris-glycine buffers, suggesting that the higher-pH suspensions interfered with MS2 recovery in some way. For comparison, a previous study by Summa et al. observed 19% viral recovery when using a pH 9.5 Tris-glycine-beef extract buffer and PEG precipitation to separate and concentrate HuNoV from lettuce samples, though it should be noted they achieved only 3% recovery when using immunomagnetic separation [43]. It is possible that neutralizing the pH of the lettuce rinsate would have helped counteract this loss of recovery, but that would be difficult to perform in an in-field setting, which is the intended eventual application for MILs. Therefore, care should be taken when selecting an elution buffer to release viral particles from food samples in preparation for MIL-based capture. However, these results demonstrate that, with an appropriate viral elution buffer, MILs can show highly favorable capture and recovery efficiency even in complex suspensions with significantly reduced time and equipment requirements compared with existing sample preparation methods. Additionally, since only 1 mL of eluate was processed in this study, exploring methods for scale-up could be a fruitful area for further study. These could include increasing input volume, successive target binding steps, or reducing the relative volume of elution buffer to target sample.

As mentioned above, MILs are non-specific binding reagents, which can be advantageous when targeting viruses as genetically diverse as HuNoV, but can also present challenges when used with complex matrices. Food samples or the slurries and dilutions prepared from them may contain components that could compete for binding space on the surfaces of MIL droplets or inhibit endpoint detection by RT-PCR or other nucleic acid amplification methods. However, the results presented herein suggest that MILs may find advantageous and highly relevant applications for analyses of produce washes (leafy greens, fruit, vegetables), as well as spent seed sprout irrigation water and similar lower-complexity systems [44]. These are just some of the matrices in which HuNoV and other viruses are of major concern, so further study in other complex matrices will be necessary, but this work shows promising initial results. Additionally, favorable results in previous studies for MIL-based capture and concentration of bacteria from liquid food matrices suggest that similar outcomes may be possible with other electrostatically charged analytes, such as viruses and viral ssRNA [24].

## Conclusions

Our results demonstrate the utility of hydrophobic MILs as novel biocompatible target separation solvents for both non-enveloped viruses and viral genomic ssRNA in aqueous suspension. Further study is needed for transition metal-based MILs, particularly regarding the effects of suspension characteristics such as pH and solute concentrations on MIL dispersion and target binding, as well as enrichment capacity. Additionally, different elution methods should be explored with rare earth metal-based MILs to determine if target recovery closer to the observed capture efficiency can be achieved.

In 2019, the National Academies of Sciences, Engineering and Medicine released a report entitled “A Research Agenda for Transforming Separation Science.” The report outlines an ambitious agenda for future research in separation science, targets critical areas for greater focus, and notes the vital importance of cross-disciplinary work to strengthening national capabilities in separation science. Key areas identified in the report that are primed for collaborative exploration by analytical chemists and microbiologists include development of approaches for separating multicomponent mixtures, separation of dilute analytes and leveraging multiple forces, or cooperative binding mechanisms for improved separations. To date, MILs have been used for separation of various chemical, biomolecular, and microbiological analytes from clinical, environmental, or food matrices. The present work further underlines the promise of MILs as broadly applicable sample preparation reagents useful for analytes spanning molecular, viral, and cellular scales and compatible with multiple methods for downstream analysis (i.e., culture, plaque assay, nucleic acid amplification) [45]. Given the requirement for only a magnet and their potential to be used both for particle capture followed by genomic nucleic acid extraction, MILs could have promise for application in in-field detection settings with minimal use of equipment. Alternatively, this property also suggests that MILs could also have value as a reagent in automated or semi-automated workflows in settings where more equipment and resources are available as well. For example, Hice et al. suggested the use of a strong electromagnet, which could be operated in the context of an automated multi-well plate assay, for high-throughput manipulation of MIL-target complexes prior to downstream analyses [46].

Overall, the present work provides a foundation for future studies of MIL-based viral capture and concentration and RNA extraction, gives further insight into some of the factors that could influence the binding between non-enveloped viruses or viral genomic ssRNA and MILs, and demonstrates the capacity of MILs to separate and concentrate non-enveloped viral particles from a complex suspension. Further, cost is a major consideration when evaluating testing methods for foods. The authors estimate that MILs can currently be generated for about $0.50–0.70 per milliliter, a value which could be further reduced by evaluating the potential to recycle MILs for reuse despite their already comparatively low cost [46–48]. Though there remain many potential areas for further study, this proof-of-concept work suggests that MILs have potential to become a valuable technology for virus capture, concentration, and detection in-field and in food preparation settings, enabling faster and more cost-effective microbial testing for food producers and processors.

## References

[1] Kim S-O, Kim S-S. Recent (2011–2017) foodborne outbreak cases in the Republic of Korea compared to the United States: a review. Food Sci Biotechnol. 2021;30:185–94. 10.1007/s10068-020-00864-x. 33732509 10.1007/s10068-020-00864-x PMC7914323

[2] Scharff RL. Economic burden from health losses due to foodborne illness in the United States. 10.4315/0362-028X.JFP-11-058. 10.4315/0362-028X.JFP-11-058 22221364

[3] Hoffmann S, Batz MB, Morris JG. Annual cost of illness and quality-adjusted life year losses in the United States due to 14 foodborne pathogens. J Food Protect. 2012;75:1292–302. 10.4315/0362-028X.JFP-11-417. 10.4315/0362-028X.JFP-11-417 22980013

[4] Teunis PFM, Moe CL, Liu P, Miller SE, Lindesmith L, Baric RS, Le PJ, Calderon RL. Norwalk virus : how infectious is it ? J Med Virol. 2008;80:1468–76. 10.1002/jmv. 18551613 10.1002/jmv.21237

[5] Teunis PFM, Le Guyader FS, Liu P, Ollivier J, Moe CL. Noroviruses are highly infectious but there is strong variation in host susceptibility and virus pathogenicity. Epidemics. 2020;32:100401. 10.1016/J.EPIDEM.2020.100401. 32721875 10.1016/j.epidem.2020.100401

[6] Girard M, Ngazoa S, Mattison K, Jean J. Attachment of noroviruses to stainless steel and their inactivation, using household disinfectants. J Food Protect. 2010;73:400–4. 10.4315/0362-028x-73.2.400 20132692

[7] Djebbi-Simmons D, Alhejaili M, Janes M, King J, Xu W. Survival and inactivation of human norovirus GII.4 Sydney on commonly touched airplane cabin surfaces. AIMS Public Health. 2020;7:574–86. 10.3934/publichealth.2020046. 32968679 10.3934/publichealth.2020046 PMC7505796

[8] Kamarasu P, Hsu H-Y, Moore MD. Research progress in viral inactivation utilizing human norovirus surrogates. Front Sustain Food Syst. 2018;2:89. 10.3389/FSUFS.2018.00089.

[9] Nugen SR, Baeumner AJ. Trends and opportunities in food pathogen detection. Anal Bioanalytic Chem. 2008;391:451–4. 10.1007/s00216-008-1886-2. 10.1007/s00216-008-1886-2 PMC2386755 18347781

[10] Vidic J, Vizzini P, Manzano M, Kavanaugh D, Ramarao N, Zivkovic M, Radonic V, Knezevic N, Giouroudi I, Gadjanski I. Point-of-need DNA testing for detection of foodborne pathogenic bacteria. Sensors. 2019;19:1100. 10.3390/S19051100. 30836707 10.3390/s19051100 PMC6427207

[11] Everitt ML, Tillery A, David MG, Singh N, Borison A, White IM. A critical review of point-of-care diagnostic technologies to combat viral pandemics. Analytica Chimica Acta. 2021;1146:184. 10.1016/J.ACA.2020.10.009. 33461715 10.1016/j.aca.2020.10.009 PMC7548029

[12] Zaczek-Moczydlowska MA, Beizaei A, Dillon M, Campbell K. Current state-of-the-art diagnostics for norovirus detection: model approaches for point-of-care analysis. Trends Food Sci Technol. 2021;114:684–95. 10.1016/j.tifs.2021.06.027.

[13] Demeke T, Adams RP. The effects of plant polysaccharides and buffer additives on PCR. BioTechniques. 1992;12:332–4. 1571138

[14] Suther C, Moore MD. Quantification and discovery of PCR inhibitors found in food matrices commonly associated with foodborne viruses. Food Sci Human Welln. 2019;8:351–5. 10.1016/j.fshw.2019.09.002.

[15] Akane A, Matsubara K, Nakamura H, Takahashi S, Kimura K. Identification of the heme compound copurified with deoxyribonucleic acid (DNA) from bloodstains, a major inhibitor of polymerase chain reaction (PCR) amplification. 1994;39:62–72. 8195750

[16] Dwivedi HP, Jaykus A. Detection of pathogens in foods: the current state-of-the-art and future directions. Critical Rev Microbiol. 2011;37:40–63. 10.3109/1040841X.2010.506430. 20925593 10.3109/1040841X.2010.506430

[17] Wang SS, Canida TA, Ihrie JD, Chirtel SJ. Sample size determination for food sampling. J Food Prot. 2023;86:100134. 10.1016/J.JFP.2023.100134. 37516241 10.1016/j.jfp.2023.100134

[18] Stals A, Baert L, Van Coillie E, Uyttendaele M. Extraction of food-borne viruses from food samples: a review. Int J Food Microbiol. 2012;153:1–9. 10.1016/j.ijfoodmicro.2011.10.014. 22137685 10.1016/j.ijfoodmicro.2011.10.014

[19] Harrington PR, Vinjé J, Moe CL, Baric RS. Norovirus capture with histo-blood group antigens reveals novel virus-ligand interactions. J Virol. 2004;78:3035–45. 14990722 10.1128/JVI.78.6.3035-3045.2004 PMC353760

[20] Huang P, Farkas T, Zhong W, Tan M, Thornton S, Morrow AL, Jiang X, Harbor P. Norovirus and histo-blood group antigens : demonstration of a wide spectrum of strain specificities and classification of two major binding groups among multiple binding patterns. J Virol. 2005;79:6714–22. 10.1128/JVI.79.11.6714. 15890909 10.1128/JVI.79.11.6714-6722.2005 PMC1112114

[21] Hayashi S, Hamaguchi HO. Discovery of a magnetic ionic liquid [bmim]FeCl4. 33:1590–1591. 10.1246/CL.2004.1590.

[22] Krieger BM, Lee HY, Emge TJ, Wishart JF, Castner EW Jr. Ionic liquids and solids with paramagnetic anions. Phys Chem Chem Phys. 2010;12:8919–25. 10.1039/c001176m. 20563329 10.1039/b920652n

[23] Clark KD, Purslow JA, Pierson SA, Nacham O, Anderson JL. Rapid preconcentration of viable bacteria using magnetic ionic liquids for PCR amplification and culture-based diagnostics. Analytic Bioanalytic Chem. 2017;409:4983–91. 10.1007/s00216-017-0439-y. 10.1007/s00216-017-0439-y 28634762

[24] Hice SA, Clark KD, Anderson JL, Brehm-Stecher BF. Capture, concentration, and detection of salmonella in foods using magnetic ionic liquids and recombinase polymerase amplification. Analytic Chem. 2019;91:1113–20. 10.1021/acs.analchem.8b04751. 10.1021/acs.analchem.8b04751 30499290

[25] Clark KD, Nacham O, Yu H, Li T, Yamsek MM, Ronning DR, Anderson JL. Extraction of DNA by magnetic ionic liquids: tunable solvents for rapid and selective DNA analysis. Analytic Chem. 2015;87:1552–9. 10.1021/ac504260t. 10.1021/ac504260t 25582771

[26] Emaus MN, Clark KD, Hinners P, Anderson JL. Preconcentration of DNA using magnetic ionic liquids that are compatible with real-time PCR for rapid nucleic acid quantification. Analytic Bioanalytic Chem. 2018;410:4135–44. 10.1007/s00216-018-1092-9. 10.1007/s00216-018-1092-9 29704032

[27] Emaus MN, Anderson JL. Magnetic ionic liquids as microRNA extraction solvents and additives for the exponential amplification reaction. Analytica Chimica Acta. 2021;1181:338900. 10.1016/J.ACA.2021.338900. 34556230 10.1016/j.aca.2021.338900

[28] Clark KD, Sorensen M, Nacham O, Anderson JL. Preservation of DNA in nuclease-rich samples using magnetic ionic liquids †. RSC Adv. 2016. 10.1039/c6ra05932e.

[29] Pierson SA, Nacham O, Clark KD, Nan H, Mudryk Y, Anderson JL. Synthesis and characterization of low viscosity hexafluoroacetylacetonate-based hydrophobic magnetic ionic liquids. New J Chem. 2017;41:5498–505. 10.1039/c7nj00206h.

[30] Bae J, Schwab KJ. Evaluation of murine norovirus, feline calicivirus, poliovirus, and MS2 as surrogates for human norovirus in a model of viral persistence in surface water and groundwater. Appl Environ Microbiol. 2008;74:477–84. 10.1128/AEM.02095-06. 18065626 10.1128/AEM.02095-06 PMC2223264

[31] Dawson DJ, Paish A, Staffell LM, Seymour IJ, Appleton H. Survival of viruses on fresh produce, using MS2 as a surrogate for norovirus. J Appl Microbiol. 2005;98:203–9. 10.1111/J.1365-2672.2004.02439.X. 15610433 10.1111/j.1365-2672.2004.02439.x

[32] Brandsma SR, Muehlhauser V, Jones TH. Survival of murine norovirus and F-RNA coliphage MS2 on pork during storage and retail display. Int J Food Microbiol. 2012;159:193–7. 10.1016/J.IJFOODMICRO.2012.09.015. 23107497 10.1016/j.ijfoodmicro.2012.09.015

[33] Su X, Zivanovic S, D’Souza DH. Effect of chitosan on the infectivity of murine norovirus, feline calicivirus, and bacteriophage MS2. J Food Protect. 2009;72:2623–8. 10.4315/0362-028x-72.12.2623. 10.4315/0362-028x-72.12.2623 20003751

[34] Chomczynski P, Sacchi N. Single-step method of RNA isolation by acid guanidinium thiocyanate-phenol-chloroform extraction. Anal Biochem. 1987;162:156–9. 10.1016/0003-2697(87)90021-2. 2440339 10.1006/abio.1987.9999

[35] O’Connell KP, Bucher JR, Anderson PE, Cao CJ, Khan AS, Gostomski MV, Valdes JJ. Real-time fluorogenic reverse transcription-PCR assays for detection of bacteriophage MS2. Appl Environ Microbiol. 2006;72:478–83. 10.1128/AEM.72.1.478-483.2006. 16391081 10.1128/AEM.72.1.478-483.2006 PMC1352182

[36] Cormier J, Janes M. A double layer plaque assay using spread plate technique for enumeration of bacteriophage MS2. J Virol Methods. 2014;196:86–92. 10.1016/J.JVIROMET.2013.10.034. 24211298 10.1016/j.jviromet.2013.10.034

[37] Ha JH, Choi C, Do Ha S. Evaluation of immunomagnetic separation method for the recovery of hepatitis A virus and GI.1 and GII.4 norovirus strains seeded on oyster and mussel. Food Environ Virol. 2014;6:290–6. 10.1007/S12560-014-9156-2/TABLES/2. 24952877 10.1007/s12560-014-9156-2

[38] Raymond P, Paul S, Perron A, Deschênes L, Hara K. Extraction of human noroviruses from leafy greens and fresh herbs using magnetic silica beads. Food Microbiol. 2021;99:103827. 10.1016/j.fm.2021.103827. 34119112 10.1016/j.fm.2021.103827

[39] Raymond P, Paul S, Perron A, Deschênes L. Norovirus extraction from frozen raspberries using magnetic silica beads. Food Environ Virol. 2021;13:248–58. 10.1007/S12560-021-09466-0. 33651330 10.1007/s12560-021-09466-0 PMC8116234

[40] Fister S, Fuchs S, Mester P, Kilpeläinen I, Wagner M, Rossmanith P. The use of ionic liquids for cracking viruses for isolation of nucleic acids. Sep Purific Technol. 2015;155:38–44. 10.1016/j.seppur.2015.03.035.

[41] Zhu C, Varona M, Anderson JL. Magnetic ionic liquids as solvents for RNA Extraction and Preservation. ACS Omega. 2020;5:11151–9. 10.1021/ACSOMEGA.0C01098/ASSET/IMAGES/LARGE/AO0C01098_0005.JPEG. 32455238 10.1021/acsomega.0c01098 PMC7241037

[42] Hice SA, Varona M, Brost A, Dai F, Anderson JL, Brehm-Stecher BF. Magnetic ionic liquids: interactions with bacterial cells, behavior in aqueous suspension, and broader applications. Anal Bioanal Chem. 2020. 10.1007/s00216-020-02457-3. 32043203 10.1007/s00216-020-02457-3

[43] Summa M, von Bonsdorff C-H, Maunula L. Evaluation of four virus recovery methods for detecting noroviruses on fresh lettuce, sliced ham, and frozen raspberries. J Virol Methods. 2012;183:154–60. 10.1016/j.jviromet.2012.04.006. 22580195 10.1016/j.jviromet.2012.04.006

[44] Wang Q, Hirneisen KA, Markland SM, Kniel KE. Survival of murine norovirus, Tulane virus, and hepatitis A virus on alfalfa seeds and sprouts during storage and germination. Appl Environ Microbiol. 2013;79:7021–7. 10.1128/AEM.01704-13. 24014537 10.1128/AEM.01704-13 PMC3811553

[45] Moore MD, Bisha B, Anderson J, Brehm-Stecher B. Sample preparation for detection of microbiological and chemical analytes. In: Encyclopedia of Food Safety. Academic Press 2024;285–294.

[46] Hice SA, Clark KD, Anderson JL, Brehm-Stecher BF. Capture, concentration, and detection of *Salmonella* in foods using magnetic ionic liquids and recombinase polymerase amplification. Anal Chem. 2019;91:1113–20. 10.1021/acs.analchem.8b04751. 30499290 10.1021/acs.analchem.8b04751

[47] Khoo YS, Tjong TC, Chew JW, Hu X. Techniques for recovery and recycling of ionic liquids: a review. Sci Total Environ. 2024;922:171238. 10.1016/J.SCITOTENV.2024.171238. 38423336 10.1016/j.scitotenv.2024.171238

[48] Taylor AW, Lovelock KRJ, Deyko A, Licence P, Jones RG. High vacuum distillation of ionic liquids and separation of ionic liquid mixtures. Phys Chem Chem Phys. 2010;12:1772–83. 10.1039/B920931J. 20145842 10.1039/b920931j

